# Groundwater, soil moisture, light and weather data collected in a coastal forest bordering a salt marsh in the Delmarva Peninsula (VA)

**DOI:** 10.1016/j.dib.2022.108584

**Published:** 2022-09-11

**Authors:** Giovanna Nordio, Sergio Fagherazzi

**Affiliations:** Department of Earth and Environment, Boston University, United States

**Keywords:** Forest hydrology, Coastal hydrology, Conductivity, Water content, External inputs

## Abstract

Data presented in this paper were collected in eight sites across a coastal forest in the Delmarva Peninsula, VA USA. The sites, located along transects from the marshland to the inner forest, are representative of the progressive forest retreat and the consequent marsh expansion driven by sea level rise. The sites are divided in marsh, transition zone where marsh vegetation is invading the forest, low forest, where tree dieback is widespread, intermediate forest (medium forest), where trees show signs of stress, and high forest, where trees are healthy. Sea level rise and storm surge events are the drivers of the forest conversion to salt marsh. Groundwater level and electrical conductivity were measured in a well at each site. Soil water content and electrical conductivity data were measured in the first 7-cm layer of soil. Weather and light data were collected to determine the effects of external inputs on groundwater and soil moisture datasets and to relate hydrological variables and illuminance to local ecology. Data collected are fundamental to estimate feedbacks between hydrology and ecology in the study area and to quantify forest retreat due to flooding and salinization.

**Specifications Table**

**Table:** 

Subject	Environmental Science
Specific subject area	Hydrology
Type of data	Figure
How the data were acquired	Groundwater pressure, specific electrical conductivity (at 25°C) and temperature were collected using Van Essen CTD- Divers. Soil water content, specific electrical conductivity (at 25°C) and temperature were collected using Meter Group TEROS 12 sensors. Weather data were collected using a Meter Group ATMOS 41 sensor. Illuminance and temperature data were collected using Onset HOBO light sensors.
Data format	Raw, Analyzed, Filtered
Description of data collection	Groundwater and soil moisture data collection in the forest sites started in January 2019. In the marsh area, we started collecting groundwater data in January 2019 and soil moisture data in June 2021. Data collection in the transition zone started in June 2021. Data are collected every hour. Groundwater pressure is converted in groundwater level on NAVD88, knowing atmospheric pressure and soil elevation. Weather data collection started in January 2019. Precipitation, solar radiation, lightning activity, lightning distance, wind direction, wind speed, gust speed, air temperature, vapor pressure and atmospheric pressure are the variables measured. Data are collected every hour. Light variables are measured every two hours. Their collection started in June 2021. We are still collecting data and here we show data until May 2022.
Data source location	Institution: Boston University City/Town/Region: Nassawadox (VA) Country: Virginia Latitude and longitude for collected samples/data: 37°27′ North latitude and 75°50′ East longitude
Data accessibility	Data weather described in this paper can be found in: Repository name: VCR-LTER Data identification number: VCR22344 Direct URL to data: https://doi.org/10.6073/pasta/942a5a981e6e986c5fa1a9a9cd2eb8b7

## Value of the Data

• This dataset characterizes groundwater and soil moisture gradients in the coastal forest-salt marsh ecotone. Coastal forest retreat and salt marsh expansion are the consequences of salinization and flooding events driven by sea level rise and storm surges. Our data show the response of groundwater and soil moisture to hydrological drivers both at long and short timescales.
• Salt intolerant coastal trees, affected by saltwater intrusion and frequent flooding, progressively die, leaving space to more salt-tolerant vegetation. The ecological zonation at the forest boundary is visible. Our data can be used to quantify processes in the critical zone and feedbacks governing hydrology and ecology at the coast.
• These complex long-term datasets allow to study seasonal trends of hydrological variables, and to identify external drivers, like evapotranspiration and precipitation, affecting groundwater hydrology.
• Light data are benchmarks for ecological change. They can be used to investigate canopy evolution. In a healthy forest, where tree canopy is dense, the light reaching the soil is lower, while it increases after forest dieback. These data can support ecological studies on forest succession and biodiversity.
• Groundwater and soil moisture data, along with weather data, could be used for hydrological modelling, or to conduct a global analysis comparing different coastal areas.

## 1 Data Description

**Fig. 1:**
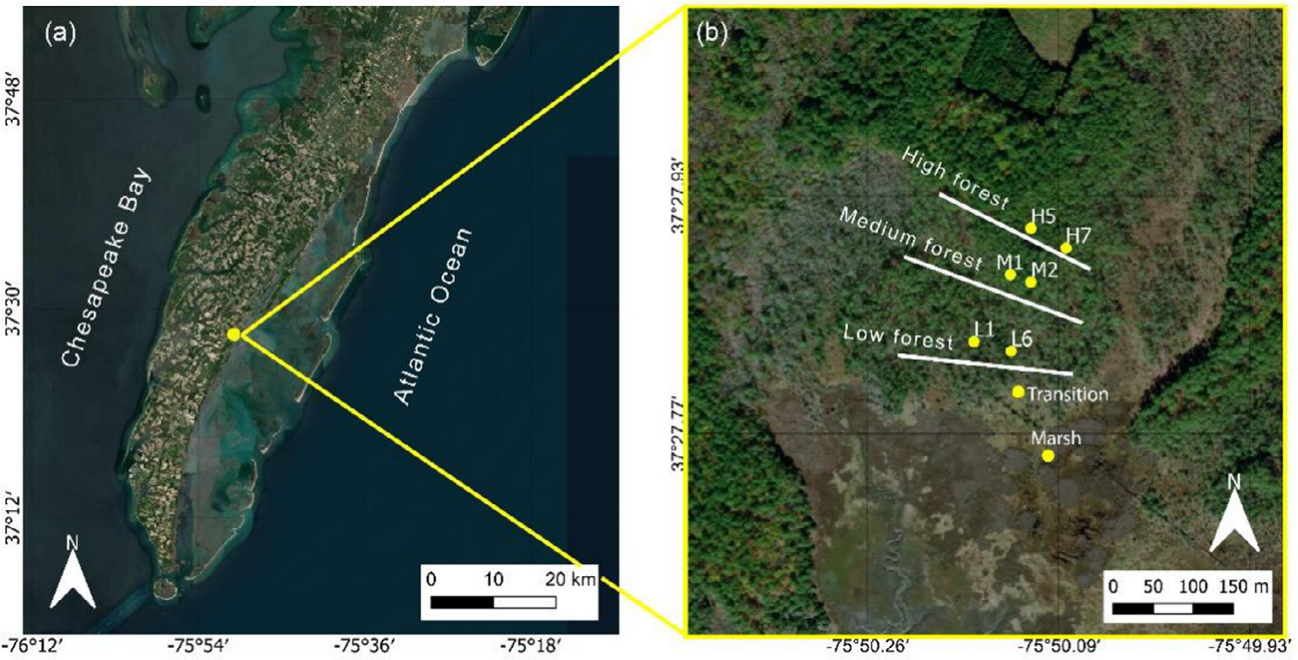
Study site in Nassawadox (VA) (a). Stations in the study site where data are collected (b).

In January 2019 the VCR-LTER program started a long-term project to develop a predictive understanding of the response of a coastal forest to sea level rise and climate change, and to relate these to the ecological services the coastal barrier systems provide [1]. In particular, the research focuses on slow environmental changes interacting with short-term disturbances, like storm events [1]. The study area is a coastal forest (Brownsville Forest) bordering a salt marsh, in Nassawadox, Delmarva Peninsula, VA, USA (Fig. 1a). The closest NOAA (National Oceanic and Atmospheric Administration) station to the study site is Wachapreague (VA) (id: 8631044). This station can be used as reference for sea level conditions. Tidal signal is semidiurnal with an amplitude ranging from around 0.8 m and 1.8 m during spring and neap tides. Storm surge events frequently occur, due to tropical storms, mostly during the fall season, and Nor'Easter storms during winter. The last storm surge events recorded in the dataset are: Melissa tropical storm, which occurred between October 11-14, 2019, when sea level reached 1.42 m on NAVD88 at the Wachapreague NOAA station (id:8631044); two storm surges occurred in May 30 and October 10, 2021, when sea level respectively reached 1.39 m and 1.37 m on NAVD88; Tropical storm Wanda, which occurred between October 26 and November 7, 2021, and was partially felt by the sensors.

The forest is dominated by *Pinus taeda*. The first layer of soil (1m) is 90% clayey and the site elevation is on average 1 m on NAVD88. In Table 1, I show more detailed information about the soil composition in each forested site. According to measures carried out with a constant head permeameter, we estimated hydraulic conductivity values ranging from 5.97 × 10^−4^ cm/min to 10.29 × 10^−4^ cm/min. To examine the hydrological gradient from marsh to forest we selected six stations along two transects, three for each transect, and we also added two sites, one in the transition zone and one in the marsh (Fig. 1b). Each site is affected by salinization and flooding events in different ways, based on its distance from the marsh boundary and elevation [2,3]. The innermost forest station presents healthy trees (sites H5, H7), the medium forest experiences some dieback and it is partially invaded by *Myrica cerifera* shrubs (sites M1, M2), the low forest is characterized by widespread tree dieback and new invasive more salt-tolerant vegetation like *Phragmites australis* (sites L1, L6), the transition zone represents the transition between forest vegetation and marsh vegetation and the marsh is dominated by *Spartina patens* and *Juncus romerianus* [4]. According to previous studies, *Pinus taeda* is moderately salt tolerant [5]. Seedling and germination are the stages most sensitive to salinization and flooding events. When groundwater levels are low, *Pinus taeda* seedlings can survive up to 5 ppt, while when groundwater tables are high they cannot tolerate any salinity level [6,7]. Mature trees affected by flooding and salinization slow their stomatal conductance, carbon assimilation and photosynthetic activity [5]. In these conditions, they can develop different techniques to tolerate, exclude or compartmentalize salt ions in leaves or roots level [8].

**Table 1:** Characteristics of the study sites.

Site	Elevation of ground surface on NAVD88 (m)	Soil compositionAverage on depth
H5	1.028	9% veg/roots, 15% sand, 76% silt/clay
H7	0.990	8% veg/roots, 10% sand, 82% silt/clay
M1	0.964	10% veg/roots, 14% sand, 76% silt/clay
M2	0.991	8% veg/roots, 16% sand, 76% silt/clay
L1	0.977	14% veg/roots, 17% sand, 69% silt/clay
L6	1.022	8% veg/roots, 13% sand, 79% silt/clay
Transition	0.984	NA
Marsh	0.957	NA

**Fig. 2:**
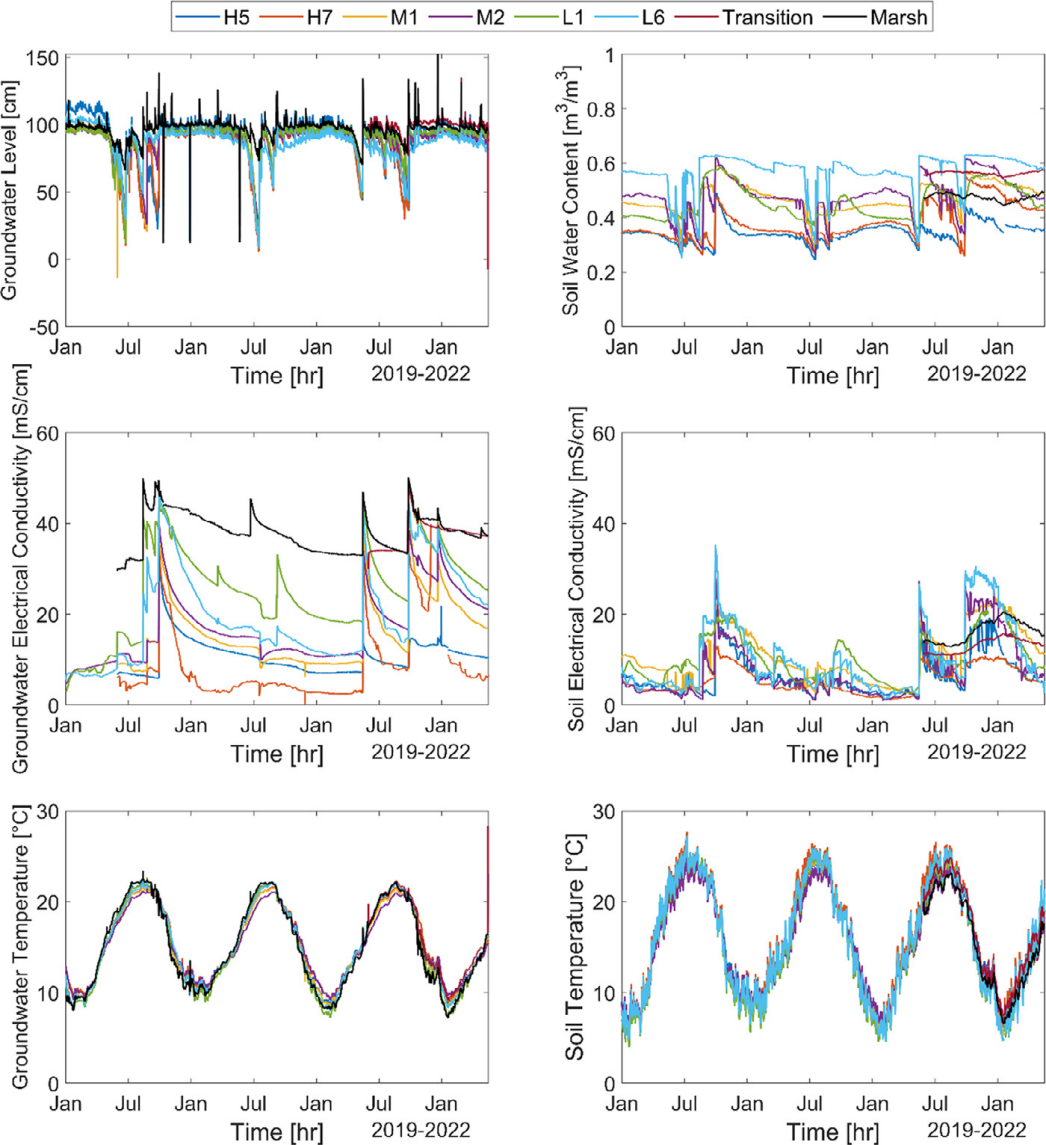
Groundwater data (left) and soil moisture data (right) collected from January 2019 to May 2022.

Here we present groundwater level (m on NAVD88), electrical conductivity (mS/cm at 25°C) and temperature (°C) data collected every hour from January 2019 to May 2022 at each site except for the transition zone where data collection started in June 2021. Groundwater data for each site are in the HS_combined.csv file in the repository and shown in Fig. 2. In the sheet we report the groundwater level data as GWLevel, groundwater temperature as GWTemperature and groundwater electrical conductivity as GWElecCond.

Soil water content (m^3^/m^3^), electrical conductivity (mS/cm at 25°C) and temperature (°C) in the first 7-cm layer of soil were collected every hour in the same time period, except for the marsh and transition zone where the soil moisture data were measured since June 2021. Soil data are reported in the HS_combined.csv file in the repository and shown in Fig. 2. In the sheet we report the soil water content data as SMWater content, the soil temperature as SMTemperature and soil electrical conductivity as SMElecCond.

**Fig. 3:**
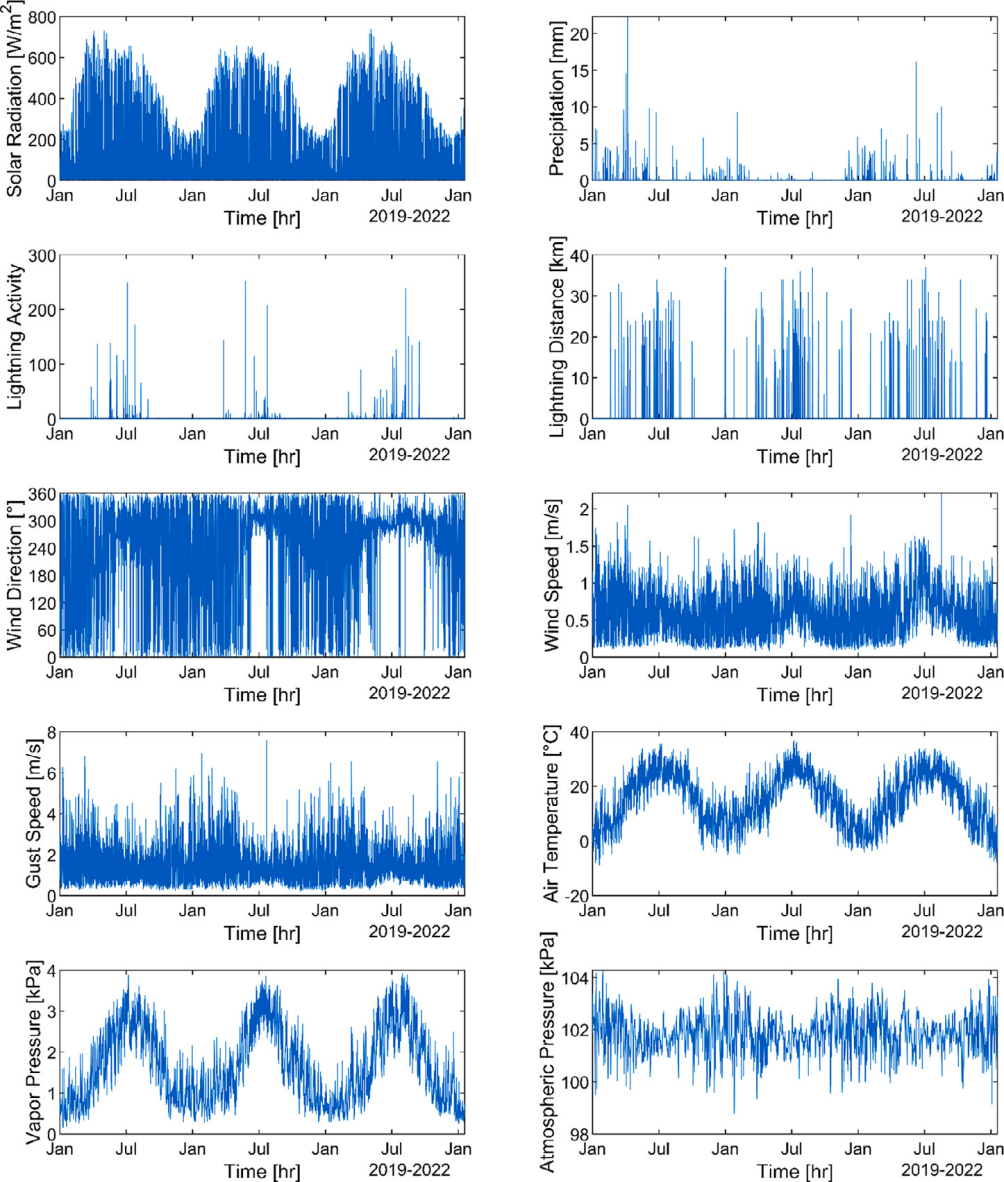
Weather data collected from January 2019 to February 2022 in M1.

Weather data collected every hour are representative of the medium forest. Precipitation (mm), solar radiation (W/m^2^), lightning activity (unitless), lightning distance (km), wind direction (°), wind speed (m/s), gust speed (m/s), air temperature (°C), vapor pressure (kPa) and atmospheric pressure (kPa) are the variables measured. Here, we present data for M1 from January 2019 to February 2022. All the variables are in the HS_Weatherdata.csv file in the repository and shown in Fig. 3.

**Fig. 4:**
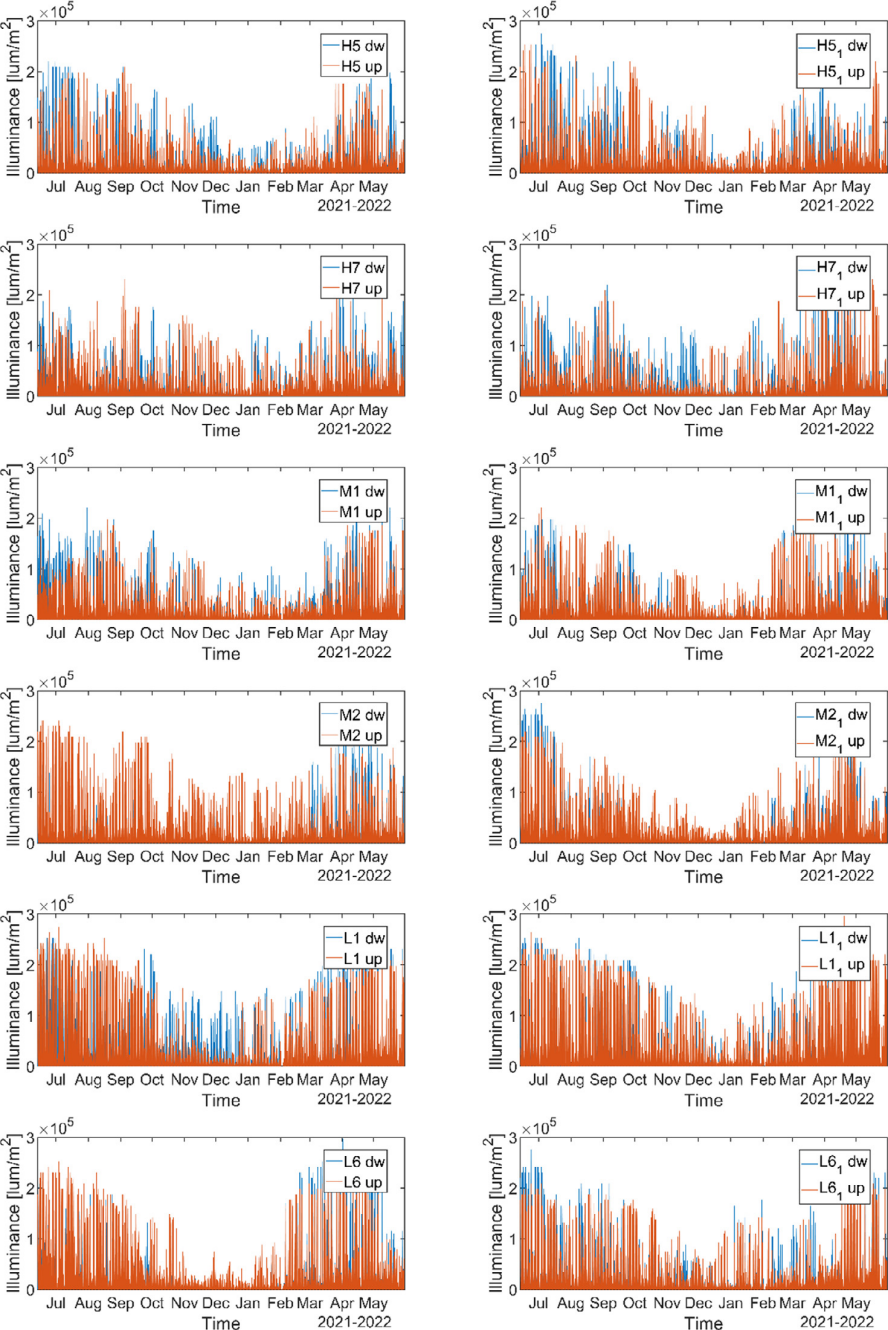
Illuminance data collected from June 2021 to May 2022. Note: dw=sensor at 30 cm above the ground surface, up= sensor at 2 m above the ground surface.

**Fig. 5:**
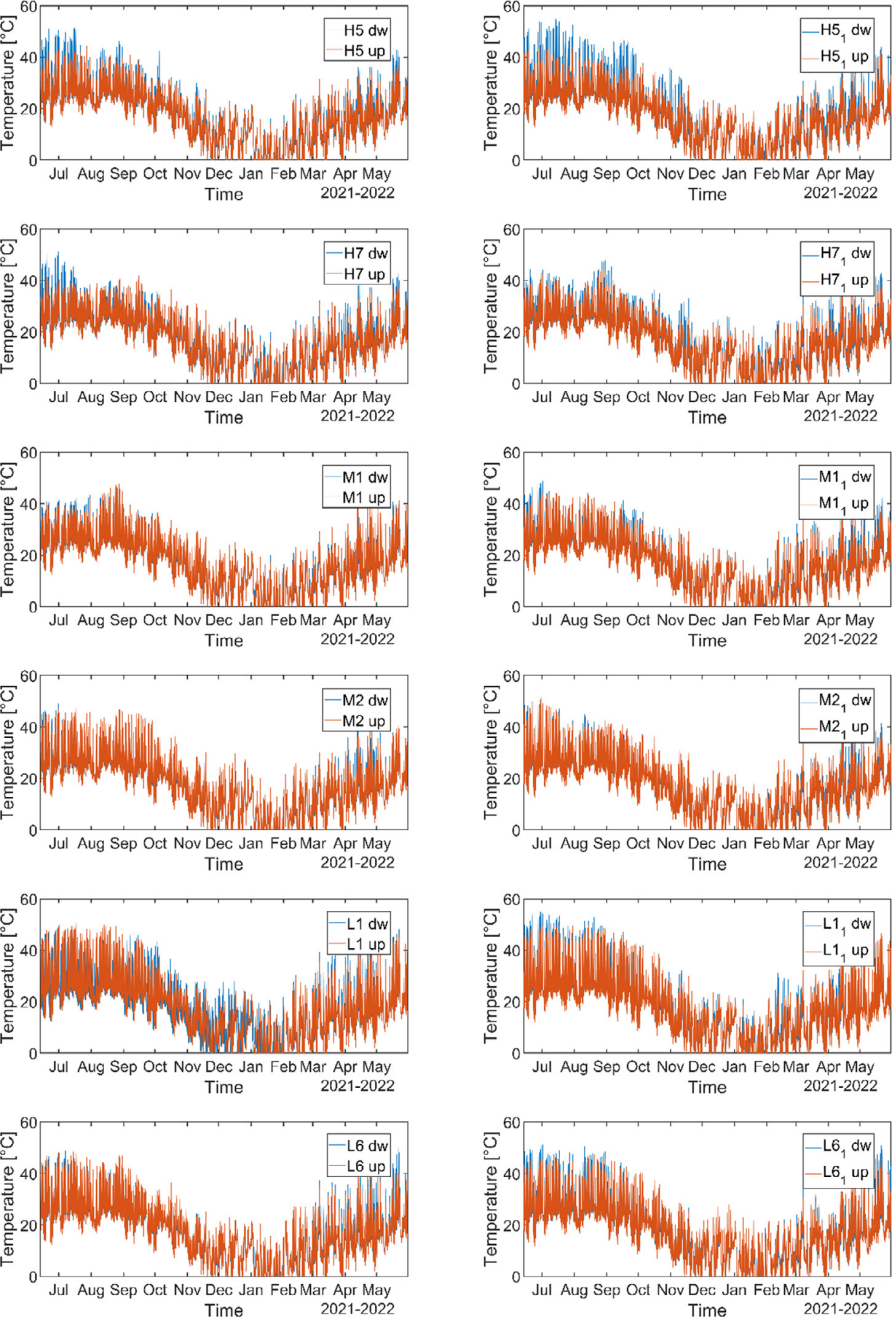
Temperature data collected from June 2021 to May 2022. Note: dw=sensor at 30 cm above the ground surface, up= sensor at 2 m above the ground surface.

Light sensors collect data of illuminance (lum/m^2^) and temperature (°C) every two hours. They started collection in June 2021. Light data are reported in the HS_ Lightdata.csv file in the repository and shown in Figs. 4 and 5. In the sheet we report the illuminance and temperature data at different heights for each forested site. Sensors at the same height in the same site are called as ‘Site name’ and ‘Site name_1’.

## 2 Experimental Design, Materials and Methods

Groundwater data were collected using CTD-Diver sensors by Van Essen. In January 2019 we installed six wells in the forested areas and one well in the marsh. In June 2021, we installed an additional well in the transition zone. The wells, screened for their entire length, were installed around 1 m below the ground surface. The sensor was hung on a cable fixed to their top closing cap. Pressure of the water column above the sensor was directly measured by the instrument. This value was firstly compensated, using atmospheric pressure from the weather station and then converted to water level on NAVD88 using georeferenced soil elevation [9]. For each well in the study site ground surface elevation data are shown in Table 1. Elevation data were collected using a Real-Time Kinematic instrument (RTK). Specific electrical conductivity and temperature did not need to be converted [9].

Soil moisture data were collected using TEROS 12 devices by Meter group. The sensors were placed 7 cm below the ground surface [10]. Weather data were collected by an ATMOS 41 sensor by Meter group. The instrument was mounted on a 2 m pole, levelled and directed to the true North [11]. Both of these sensors were connected to a ZENTRA (ZL6) data logger by Meter group, mounted on a pole and oriented toward South, to capture the maximum amount of sunlight and recharge the batteries. Data were downloaded twice per year using the ZENTRA Utility app. Particular attention was paid to clean the ATMOS 41 sensors, in its funnel part. Here leaves and organic matter tends to accumulate increasing the error in the measurements.

Light sensors were horizontally fixed on poles at two different heights. The sensors were placed horizontally on two brackets oriented toward South and perpendicularly attached to poles [12]. One sensor was placed at 30 cm above the ground surface and one at 2 m above the ground surface. Two poles are installed at each forested site.

## Ethic Statements

NA.

## CRediT Author Statement

**Giovanna Nordio:** Writing, Data collection, Visualization, Conceptualization; **Sergio Fagherazzi:** Writing, Supervision, Data collection, Conceptualization.

## Declaration of Competing Interest

The authors declare that they have no known competing financial interests or personal relationships that could have appeared to influence the work reported in this paper.

## Data Availability

Groundwater, soil moisture, light and weather data in Brownsville forest, Nassawadox, VA, 2019-2022 (Original data) (VCR-LTER).
